# Extraction of Maxillary Central Incisors: An Orthodontic-Restorative Treatment

**DOI:** 10.1155/2014/268590

**Published:** 2014-10-16

**Authors:** Zohreh Hedayati, Maryam Zare, Fateme Bahramnia

**Affiliations:** Orthodontic Research Center, School of Dentistry, Shiraz University of Medical Sciences, Ghom Abad, Ghasrodasht, Shiraz 71866-77764, Iran

## Abstract

Malformed central incisors with poor prognosis could be candidates for extraction especially in crowded dental arches. This case report refers to a 12-year-old boy who suffered from malformed upper central incisors associated with severe attrition. Upper lateral incisors were positioned palatally and canines were rotated and positioned in the high buccal area. The patient had class II malocclusion and space deficiency in both dental arches. Due to incisal wear and malformed short maxillary central incisors and the need for root canal therapy with a major crown build-up, these teeth were extracted. The maxillary lateral incisors were substituted. Thus the maxillary canines were substituted for lateral incisors and the first premolars were substituted for canines. In the lower dental arch the first bicuspids were extracted. Composite resin build-up was performed on the maxillary lateral incisors and canines. This allowed for the crowding and the malocclusion to be corrected. Subsequent gingivectomy improved the patient's gingival margins and smile esthetics one month after orthodontic therapy.

## 1. Introduction

There are many acquired and inherited developmental abnormalities that alter the size, shape, and number of teeth. Malformed teeth are derived from a developmental disturbance during odontogenesis [1].

Extraction of upper central incisors is not common in orthodontics. However, malformed central incisors with poor prognosis could be candidates for extraction [2]. The type of occlusion, space requirements, shape, size, and root height of lateral incisors and canines play important role in making decision between orthodontic and prosthodontic treatments after extraction of central incisors [3–8]. The principal approach to resolution of such problems, especially in crowded dental arches, would be orthodontic treatment and closure of anterior space by substitution of the maxillary lateral incisors. When orthodontic treatment is the choice, extraction of maxillary central incisors may provide the space to correct crowding or an increased overjet without a need for extraction of other posterior teeth [3].

In the orthodontic approach, there are some challenges: lateral incisors usually have short and tapered crown emergence profile. Periodontal deterioration may result from overcontoured mesial and distal margins of final restoration and finally there are height discrepancies between the gingival margins of lateral incisors and canines [3].

Care should be taken to

- parallel the roots of lateral incisors,
- reduce the prominence of canine root by creating a lingual torque,
- rotate the first premolars slightly in mesiopalatal direction,
- reduce palatal cusp to resemble canine [3, 4].

The presented case is a description of a class I malocclusion complicated by malformed maxillary central incisors with severe attrition and crowded dentition treated with a combined orthodontic-restorative approach.

## 2. Case Report

The patient was a twelve-year-old boy with good physical health. He had a symmetric face with a convex soft tissue profile. His chief complaint concerned malaligned and malformed anterior teeth. The patient also had a history of meningitis in his childhood.

**Figure 1:**
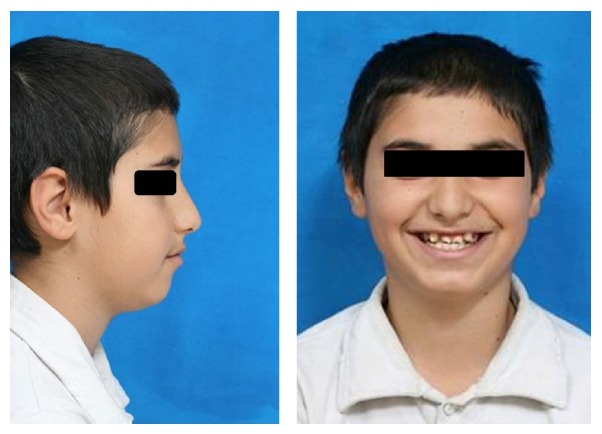
Pretreatment facial photography.

Extraoral examination showed increased vertical skeletal proportions, increased lower facial third, leptoprosopic facial type, and nonconsonant smile (Figure 1).

**Figure 2:**
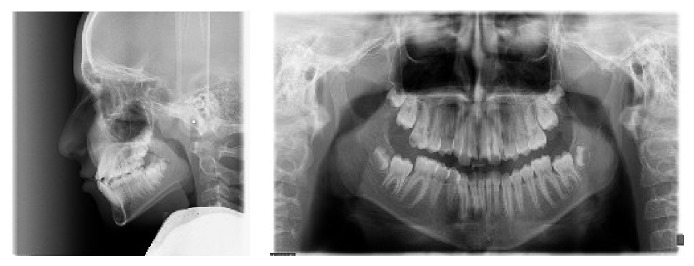
Pretreatment lateral cephalometry and panoramic radiographs.

Cephalometric analysis revealed an anteroposterior class I skeletal relationship (Figure 2). The Sum-of-Bjork and Frankfurt mandibular plane angles were both high, indicating a vertical growth pattern. Inclination of the maxillary incisors was within the normal range (102 degrees), and the mandibular incisors were proclined (96 degrees).

The patient presented with a class I malocclusion in early permanent dentition, 0 mm overjet (edge to edge bite on central incisors) and incomplete overbite in the centric occlusion, super class I molar relationship in the right side and class I molar relationship in the left side, malformed upper central incisors with incisal attrition, palatally positioned upper lateral incisors, and high buccal and rotated canines. There were 8.4 mm and 3 mm space deficiency in his upper and lower dental arches, respectively. His periodontal status was good. While his upper left primary canine was retained, all of the permanent teeth were erupted with the exception of the second and third molars.

**Figure 3:**
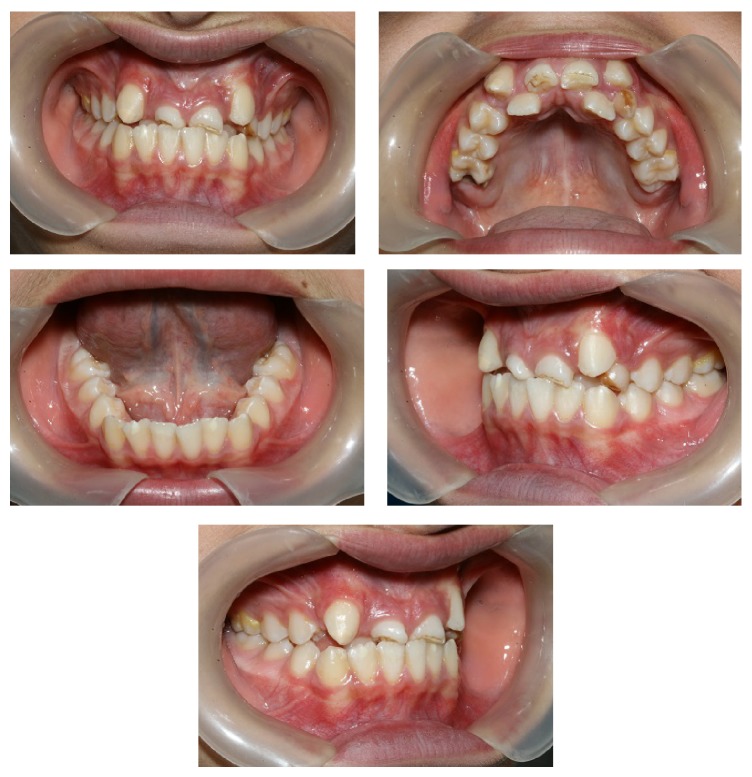
Pretreatment intraoral photography.

Lateral maxillary incisors were placed palatally with large clinical crowns and long roots. Both maxillary central incisors were malformed with short roots (Figure 3) and root canal therapy was needed due to severe attrition. Hence, from endodontic and restorative points of view, the presence of severe incisal attrition (which had led to shortening of clinical crowns), and the need for root canal therapy with a major crown build-up, central incisors were chosen as a better choice for extraction in this case.

## 3. Treatment Objectives

The objectives of the orthodontic treatment were

- producing satisfactory esthetic results by eliminating maxillary anterior crowding and mandibular anterior dental protrusion,
- correcting crossbite,
- preserving class I molar relationships,
- establishing a stable occlusion with normal overbite and overjet and esthetic smile.

## 4. Treatment Concept

Because of the above-mentioned clinical and radiographic findings, together with poor prognosis of upper central incisors and the appropriate size of lateral incisors with long roots, extraction of the upper central incisors plus substitution of the lateral incisors was determined as a suitable treatment. This involved extraction of upper central incisors to disperse the crowding in the maxilla and extraction of lower first premolars to achieve normal incisors inclination and normal overjet.

## 5. Treatment Progress

After extraction of permanent maxillary central incisors and mandibular first premolars, a preadjusted edgewise 0.019′′ by 0.022′′ slot fix appliance was placed in the maxillary and mandibular dental arches. Conventional aligning and leveling were performed. Initially, a 0.014 inch round nickel-titanium (Ni-Ti) archwire was ligated followed by a 0.016 inch round nickel-titanium. The correction of palatally lateral incisors was started by using bite raisers in order to facilitate crossing the teeth.

Once the maxillary lateral incisors had been situated in the central incisor and the maxillary canines in the lateral incisor positions, rectangular stainless steel arch wires were ligated in place to correct the torque in both arches and uprighting of the incisors roots.

**Figure 4:**
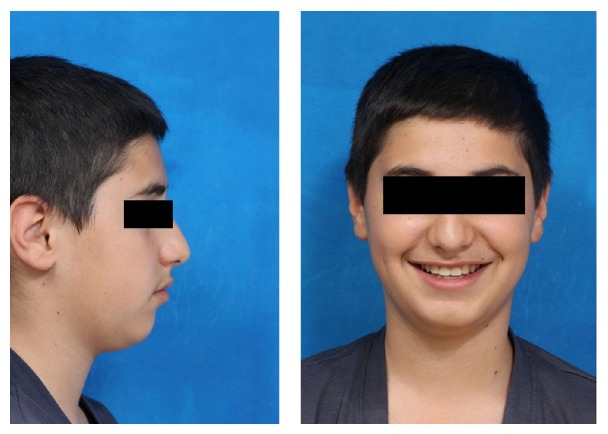
Posttreatment facial photography.

**Figure 5:**
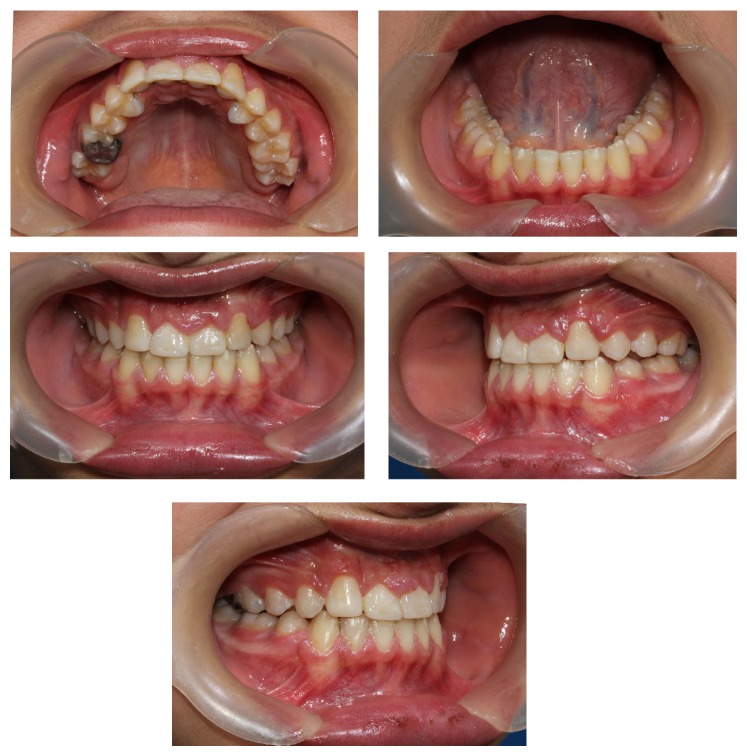
Posttreatment intraoral photographs.

The active orthodontic treatment was completed in 16 visits over the course of 19 months. At the completion of orthodontic treatment, the smile was consonant and the palatally lateral incisors were corrected. Further aims of treatment including preservation of class I molar relationships and creation of normal overjet and overbite were also achieved (Figures 4 and 5). By the completion of orthodontic treatment brackets were removed and the patient was referred for prosthodontic alteration of the shape of teeth. The maxillary lateral incisors were built up with Z100-3M resin composite to resemble central incisors. The cusps of canines were grinded. The distal and labial surfaces were flattened and reshaped to mimic lateral incisors and also meet the patient's esthetic requirements. The palatal cusps of the first bicuspids were grinded as well to make these teeth ready to serve as canines. However, an inflamed and enlarged gingival contour was present at the end of orthodontic and restorative period (Figure 5).

**Figure 6:**
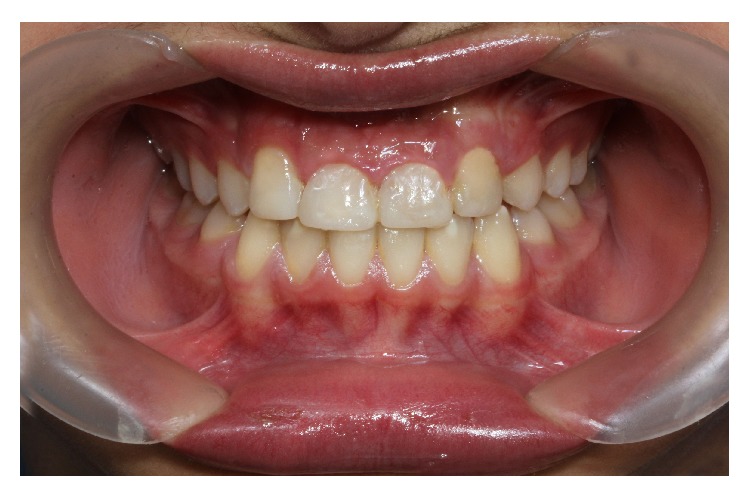
Gingival appearance after gingivectomy.

**Figure 7:**
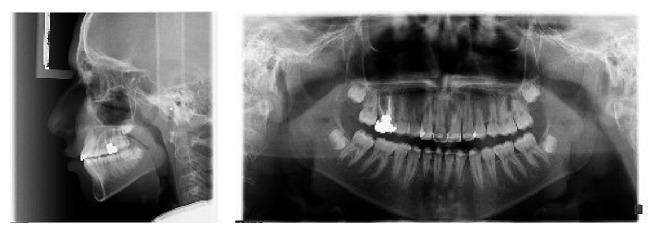
Posttreatment lateral cephalometry and panoramic radiographs.

A maxillary fixed retainer was placed following treatment to prevent any tendency for posttreatment space opening. One month later a limited gingivectomy was performed in order to eliminate hyperplastic gingival tissues and improve gingival margins. Three months later a normal gingival contour was established. However, in spite of extrusion of canines, higher levels of their gingival margin were still present (Figure 6). Normal relationship of jaw bases and dentition was present at the end of treatment (Figure 7).

## 6. Discussion

The treatment of children with poor prognosis or avulsed upper central incisors is a great challenge in dentistry. There are many approaches available to solve this problem including osseointegrated implants [4, 5], fixed or removable partial dentures, autotransplantation of other growing permanent teeth or buds [6], and orthodontic space closure. Orthodontic management by substitution of the lateral incisors for the lost or extracted central incisor teeth has been performed in many clinical experiments [3, 7–9].

The illustrated case essentially had a class I malocclusion complicated by palatally upper lateral incisors and malformed upper central incisors with severe attrition.

Clinical and radiographic assessment of upper central incisors revealed poor long-term prognosis. Therefore, they became candidate for extraction in this patient. Long roots and large crowns of the lateral incisors made these teeth appropriate substitutes for the central incisors.

Consequently, by removal of the maxillary central incisors, crowding of the upper arch was dispersed. Extraction of the lower first premolars was undertaken to create a normal overjet.

Dental esthetics was enhanced by selective incisal reduction of the maxillary canines; remodeling was performed sequentially and under cooling to avoid short-term sensitivity and long-term complications, including sclerosis [10, 11].

Mandibular excursions were also smoothed without nonworking side interferences. The prevalence of nonworking side interferences and overall temporomandibular joint health is almost identical in subjects treated with orthodontic space closure or prosthetic replacement with absent lateral incisors [12, 13]. Therefore, central incisor substitution is also unlikely to have a prolonged influence on temporomandibular integrity [14].

Utilizing the above approach is ideal when a patient is young and without gingival display in smiling. Crowding in upper dental arch or large overjet requiring extraction, lateral incisors with large clinical crowns and long roots, and small size of canines are other conditions required for this kind of approach [3].

Fortunately this young patient had the required conditions including suitable size of lateral incisors and canines, enough long roots of lateral incisors, and crowding in his both upper and lower arches.

When a lateral incisor is substituted for a missing maxillary central incisor several important steps will ensure an esthetic result. First, the gingival margins of the maxillary anterior teeth must be positioned properly [15–19]. When a lateral incisor is substituted for a central one, the canine is substituted for the lateral incisor. In this situation, the orthodontist must disregard the incisal edges of these teeth as guides for final tooth positioning [2].

During orthodontic treatment, the maxillary canines must be extruded to move their gingival margins incisally to resemble the usual gingival margin position of lateral incisors. The lateral incisors must be intruded significantly so that their gingival margins match the adjacent canines and create the illusion of normal anterior gingival levels [2]. An additional benefit of intruding the lateral incisor is to facilitate restoration of this tooth into the shape of a central incisor [15]. In this case this was done from the first stage of orthodontic treatment by altering the bracket positioning.

However, addition intrusion of the incisors could have improved the gingival margin relationships even further.

In order to enhance the gingival architecture, excessive mesial angulation of the maxillary lateral incisors (which is unwanted during space closure) should be prevented. This was avoided for the patient by placing central incisor brackets on the lateral incisors and preserving appropriate angulation. The reduced mesial angulation allowed the propensity for enhanced torque delivery. Consequently proceeding slowly with space closure to achieve ideal root positioning and using selective second-order archwire adjustments promoted mesial positioning of the lateral incisor roots.

Finally, the young patient was treated successfully. A satisfactory occlusion with coincident midlines and esthetic results was achieved.
